# Supplementary material for: Sexual behaviour and STI testing among Dutch swingers: A cross-sectional internet based survey performed in 2011 and 2018
Source: PLoS One. 2020 Oct 1;15(10):e0239750. doi: 10.1371/journal.pone.0239750 (PMC7529206; doi:10.1371/journal.pone.0239750)
Supplement: S1 File — (PDF) [file pone.0239750.s001.pdf]

Swingers, Seksualiteit & Middelengebruik

1. Doet u mee?!

Geachte deelnemer,

Het online onderzoek naar seksualiteit, middelengebruik en testgedrag bij swingers is afgerond. Daarom is het niet meer mogelijk om de vragenlijst in te vullen.

Swingers, Seksualiteit & Middelengebruik

2. Swingen

* 1. Bent u ouder dan 18 jaar?

- ◯ Ja
- ◯ Nee

Swingers, Seksualiteit & Middelengebruik

3. Swingen

* 2. Maakt u deel uit van een man/vrouw stel?

- ◯ Ja
- ◯ Nee

Swingers, Seksualiteit & Middelengebruik

4. Swingen

* 3. Hebt u, als man/vrouw stel, seksuele contacten met andere stellen en/of singles?

- Ja
- Nee

Swingers, Seksualiteit & Middelengebruik

5. Swingen

* 4. Bent u single?

- Ja
- Nee

Swingers, Seksualiteit & Middelengebruik

6. Swingen

* 5. Hebt u, als single, seksuele contacten met man/vrouw stellen?

- Ja
- Nee

Swingers, Seksualiteit & Middelengebruik

7. Swinggedrag

* 6. Hoeveel keer heeft u in de afgelopen 12 maanden ongeveer geswingd?

- 0 keer per jaar
- 1 keer per jaar
- 2 keer per jaar
- 1 keer per 3 maanden
- 1 keer per maand
- 1 keer per 2 weken
- 1 keer per week
- Vaker dan 1 keer per week

Swingers, Seksualiteit & Middelengebruik

8. Swinggedrag

* 7. Hoeveel jaren swingt u al?

Swingers, Seksualiteit & Middelengebruik

9. Swinggedrag

* 8. Waar swingt u meestal?

- Bij mij of een ander thuis
- In parenclubs
- In hotels
- Tijdens (dance/house) feesten
- Tijdens swingers vakanties
- Anders, namelijk:

Swingers, Seksualiteit & Middelengebruik

10. Swinggedrag

* 9. Met wie hebt u tijdens het swingen meestal seksueel contact?

- ○ Meestal met mannen
- ○ Meestal met vrouwen
- ○ Meestal met mannen en vrouwen

Swingers, Seksualiteit & Middelengebruik

11. Swinggedrag

* 10. Met hoeveel personen hebt u meestal seksueel contact tijdens een swingdate?
(Met seksueel contact wordt fysiek contact bedoeld)

- ○ Alleen met mijn eigen partner
- ○ (Buiten mijn eigen partner om) met 1 of 2 personen
- ○ (Buiten mijn eigen partner om) met 3 tot 5 personen
- ○ (Buiten mijn eigen partner om) met 6 of meer personen

Swingers, Seksualiteit & Middelengebruik

12. Swinggedrag

* 11. Wilt u bij onderstaande stellingen aangeven of deze voor u van toepassing zijn?

	Nooit	Soms	Meestal	Altijd	Niet van toepassing
Ik gebruik een condoom bij vaginale seks met swingpartners	○	○	○	○	○
Ik gebruik een condoom bij anale seks met swingpartners	○	○	○	○	○
Ik gebruik een condoom/ beflapje bij orale seks met swingpartners	○	○	○	○	○
Ik was mijn handen na iedere seksuele handeling	○	○	○	○	○
Ik wissel het condoom tussen anaal en vaginaal	○	○	○	○	○
Ik wissel het condoom na iedere sekspartner	○	○	○	○	○
Ik wissel het condoom bij speeltjes na ieder seksueel contact	○	○	○	○	○
Ik reinig speeltjes na ieder seksueel contact	○	○	○	○	○

Swingers, Seksualiteit & Middelengebruik

13. Middelengebruik

De volgende vragen gaan over middelengebruik tijdens het swingen.

* 12. Gebruikt u wel eens potentieverhogende middelen (Kamagra, Viagra, Cialis), tijdens het swingen?

- ○ Nooit
- ○ Soms
- ○ Meestal
- ○ Altijd

Swingers, Seksualiteit & Middelengebruik

14. Middelengebruik

* 13. Gebruikt u wel eens alcohol tijdens het swingen?

- ○ Nooit
- ○ Soms
- ○ Meestal
- ○ Altijd

15. Middelengebruik

* 14. Hoeveel alcohol drinkt u ongeveer tijdens een swingavond?

- ○ Minder dan 1 glas per avond
- ○ 1 – 2 glazen per avond
- ○ 3 – 4 glazen per avond
- ○ 5 – 6 glazen per avond
- ○ Meer dan 6 glazen per avond

Swingers, Seksualiteit & Middelengebruik

16. Middelengebruik

De volgende vragen gaan specifiek over drugsgebruik

* 15. Wilt u aankruisen welke uitspraak voor u het meest van toepassing is?

Het gebruiken van drugs tijdens het swingen vind ik...

	Helemaal oneens	Oneens	Neutraal	Eens	Helemaal eens
Acceptabel	○	○	○	○	○
Plezierig	○	○	○	○	○
Onverstandig	○	○	○	○	○
Gevaarlijk	○	○	○	○	○
Spannend	○	○	○	○	○
Ongezond	○	○	○	○	○

Swingers, Seksualiteit & Middelengebruik

17. Middelengebruik

* 16. Welke mensen in uw omgeving zouden drugsgebruik goedkeuren/accepteren?

	Zeker niet	Waarschijnlijk niet	Misschien	Waarschijnlijk wel	Zeker wel
Mijn naaste familie	◯	◯	◯	◯	◯
Mijn vrienden	◯	◯	◯	◯	◯
Mijn partner	◯	◯	◯	◯	◯
Mijn swingpartners	◯	◯	◯	◯	◯

18. Middelengebruik

* 17. Welke mensen in uw omgeving zouden drugsgebruik aanmoedigen/stimuleren?

	Zeker niet	Waarschijnlijk niet	Misschien	Waarschijnlijk wel	Zeker wel
Mijn naaste familie	◯	◯	◯	◯	◯
Mijn vrienden	◯	◯	◯	◯	◯
Mijn partner	◯	◯	◯	◯	◯
Mijn swingpartners	◯	◯	◯	◯	◯

Swingers, Seksualiteit & Middelengebruik

19. Middelengebruik

* 18. Hoe moeilijk/makkelijk is het om aan drugs te komen, denkt u?

- ◯ Erg moeilijk
- ◯ Moeilijk
- ◯ Niet moeilijk/niet makkelijk
- ◯ Makkelijk
- ◯ Erg makkelijk

Swingers, Seksualiteit & Middelengebruik

20. Middelengebruik

* 19. Swingt u met mensen die drugs gebruiken?

- Nooit
- Soms
- Meestal
- Altijd

Swingers, Seksualiteit & Middelengebruik

21. Middelengebruik

* 20. Gebruikt u zelf wel eens drugs tijdens het swingen?

- Ik gebruik wel drugs tijdens het swingen
- Ik gebruik geen drugs tijdens het swingen, maar heb dit in het verleden wel gedaan
- Ik heb nog nooit drugs gebruikt tijdens het swingen

Swingers, Seksualiteit & Middelengebruik

22. Middelengebruik

* 21. Wat was voor u de belangrijkste reden om te starten met drugsgebruik?

- Ik werd nieuwsgierig omdat ik mensen in mijn omgeving zag gebruiken of hierover hoorde
- Ik kreeg drugs aangeboden
- Ik werd overgehaald om drugs te gebruiken
- Anders, namelijk:

Swingers, Seksualiteit & Middelengebruik

23. Middelengebruik

* 22. Gebruikt u wel eens XTC tijdens het het swingen?

- ◯ Ja
- ◯ Nee

Swingers, Seksualiteit & Middelengebruik

24. Middelengebruik

* 23. Hoe vaak en hoeveel XTC gebruikt u tijdens het swingen?

	Hoe vaak gebruikt u XTC	Totale hoeveelheid per avond
XTC		

Swingers, Seksualiteit & Middelengebruik

25. Middelengebruik

* 24. Gebruikt u wel eens GHB tijdens het swingen?

- ◯ Ja
- ◯ Nee

Swingers, Seksualiteit & Middelengebruik

26. Middelengebruik

* 25. Hoe vaak en hoeveel GHB gebruikt u tijdens het swingen?

	Hoe vaak gebruikt u GHB	Totale hoeveelheid per avond
GHB		

Swingers, Seksualiteit & Middelengebruik

27. Middelengebruik

* 26. Gebruikt u wel eens lachgas tijdens het swingen?

- ◯ Ja
- ◯ Nee

Swingers, Seksualiteit & Middelengebruik

28. Middelengebruik

* 27. Hoe vaak gebruikt u lachgas tijdens het swingen?

- ◯ Soms
- ◯ Meestal
- ◯ Altijd

Swingers, Seksualiteit & Middelengebruik

29. Middelengebruik

* 28. Hoeveel ballonnen lachgas gebruikt u tijdens het swingen?
(Totale aantal ballonnen per avond)

[ ]

Swingers, Seksualiteit & Middelengebruik

30. Middelengebruik

* 29. Gebruikt u wel eens speed/amfetamine tijdens het swingen?

- Ja
- Nee

Swingers, Seksualiteit & Middelengebruik

31. Middelengebruik

* 30. Hoe vaak en hoeveel speed/amfetamine gebruikt u tijdens het swingen?

	Hoe vaak gebruikt u speed/amfetamine	Totale hoeveelheid per avond
Speed / Amfetamine		

Swingers, Seksualiteit & Middelengebruik

32. Middelengebruik

* 31. Gebruikt u wel eens poppers tijdens het swingen?

- Ja
- Nee

Swingers, Seksualiteit & Middelengebruik

33. Middelengebruik

* 32. Hoe vaak gebruikt u poppers tijdens het swingen?

- Soms
- Meestal
- Altijd

Swingers, Seksualiteit & Middelengebruik

34. Middelengebruik

* 33. Hoeveel snuiven/inhalaties poppers gebruikt u tijdens het swingen?
(Totale aantal snuiven/inhalaties per avond)

Swingers, Seksualiteit & Middelengebruik

35. Middelengebruik

* 34. Gebruikt u wel eens ketamine tijdens het swingen?

- Ja
- Nee

Swingers, Seksualiteit & Middelengebruik

36. Middelengebruik

* 35. Hoe vaak en hoeveel ketamine gebruikt u tijdens het swingen?

	Hoe vaak gebruikt u ketamine	Totale hoeveelheid per avond
Ketamine		

Swingers, Seksualiteit & Middelengebruik

37. Middelengebruik

* 36. Gebruikt u wel eens cocaïne tijdens het swingen?

- Ja
- Nee

Swingers, Seksualiteit & Middelengebruik

38. Middelengebruik

* 37. Hoe vaak en hoeveel cocaïne gebruikt u tijdens het swingen?

	Hoe vaak gebruikt u cocaïne	Totale hoeveelheid per avond
Cocaïne		

Swingers, Seksualiteit & Middelengebruik

39. Middelengebruik

* 38. Gebruikt u tijdens het swingen nog andere middelen die niet benoemd zijn? (Denk hierbij onder andere aan: Cannabis, Paddo's, Heroine, LSD, Khat)

- Nee
- Ja, namelijk:

Swingers, Seksualiteit & Middelengebruik

40. Middelengebruik

* 39. Hoeveel gebruikt u hiervan tijdens het swingen?

Swingers, Seksualiteit & Middelengebruik

41. Middelengebruik

* 40. Gebruikt u wel eens meer dan 1 soort drugs tijdens een swingavond?

- ◯ Nooit
- ◯ Soms
- ◯ Meestal
- ◯ Altijd

Swingers, Seksualiteit & Middelengebruik

42. Middelengebruik

* 41. Welke typen drugs combineert u het meest/vaakst?

Swingers, Seksualiteit & Middelengebruik

43. Middelengebruik

* 42. Als u drugs gebruikt tijdens het swingen, welke POSITIEVE effecten ervaart u dan?

"Als ik drugs gebruik, dan...

	Nooit	Soms	Meestal	Altijd
a ...heb ik meer energie en kan ik langer doorgaan	○	○	○	○
b ...ben ik meer alert	○	○	○	○
c ...kan ik hardere seksuele handeling verrichten en/of laten verrichten	○	○	○	○
d ...ervaar ik een liefdevol/intiem gevoel	○	○	○	○
e ...ervaar ik een rustig / ontspannen gevoel	○	○	○	○
f ...ervaar ik een positief / gelukkig gevoel	○	○	○	○
g ...vergeet ik mijn problemen	○	○	○	○
h ...ben ik meer seksueel opgewonden	○	○	○	○
i ...ervaar ik minder remmingen / voel ik mij vrijer	○	○	○	○
j ...kan ik meer genieten omdat het mijn orgasme uitstelt	○	○	○	○
k ...kan ik meer genieten omdat het orgasme intenser wordt	○	○	○	○
l ...beleef ik licht en geluid intenser	○	○	○	○
m ...ben ik mij minder bewust van tijd en plaats	○	○	○	○
n ...leg ik makkelijker contacten	○	○	○	○
o ...raak ik makkelijker andere mensen aan en/of laat ik mij makkelijker aanraken	○	○	○	○
p ...voel ik mij, de dag(en) na het swingen prettiger	○	○	○	○

q ...anders, namelijk:

* 43. Welke 3 van bovenstaande effecten ervaart u als belangrijkste POSITIEVE effecten? Gebruik hiervoor de letters die voor de effecten staan (bovenstaande vraag)

Swingers, Seksualiteit & Middelengebruik

44. Middelengebruik

* 44. Als u drugs gebruikt tijdens het swingen, welke NEGATIEVE effecten ervaart u dan TIJDENS het swingen?
(korte termijn effecten)

“Als ik drugs gebruik, dan...

	Nooit	Soms	Meestal	Altijd
a ...kan ik 'out' gaan	◯	◯	◯	◯
b ...word ik agressief	◯	◯	◯	◯
c ...word ik emotioneel	◯	◯	◯	◯
d ...word ik angstig	◯	◯	◯	◯
e ...ga ik hallucineren	◯	◯	◯	◯
f ...heb ik minder zin in seks	◯	◯	◯	◯
g ...verricht ik seksuele handelingen die ik zonder drugs niet zou verrichten	◯	◯	◯	◯
h ...vergeet ik wel eens condooms te gebruiken	◯	◯	◯	◯
i ...krijg ik geen erectie en/of orgasme	◯	◯	◯	◯
j ...ben ik mij minder bewust van tijd en plaats	◯	◯	◯	◯

k ...anders, namelijk:

* 45. Als u drugs gebruikt tijdens het swingen, welke NEGATIEVE effecten ervaart u dan NA AFLOOP van het swingen? (lange termijn effecten)

“Als ik drugs gebruik, dan...

	Nooit	Soms	Meestal	Altijd
l ...voel ik mij misselijk	○	○	○	○
m ...krijg ik hoofdpijn	○	○	○	○
n ...ben ik, de dagen na het swingen, vermoeid	○	○	○	○
o ...heb ik steeds meer drugs nodig voor hetzelfde effect	○	○	○	○
p ...heb ik, de dagen na het swingen, last van slapeloosheid	○	○	○	○
q ...kan ik verslaafd raken	○	○	○	○
r ...schaadt dit mijn gezondheid	○	○	○	○
s ...voel ik mij, de dagen na het swingen, down/depressief	○	○	○	○
t ...vind ik het niet prettig meer om seks te hebben zonder drugs	○	○	○	○
u ...functioneer ik, de dagen na het swingen, minder goed op mijn werk	○	○	○	○
v ...heb ik, de dagen na het swingen, minder energie voor mijn gezin/kinderen	○	○	○	○

w ...anders, namelijk:

* 46. Welke 3 van bovenstaande effecten ervaart u als meest NEGATIEF? Gebruik hiervoor de letters die voor de effecten staan (bovenstaande 2 vragen)

Swingers, Seksualiteit & Middelengebruik

45. Middelengebruik

* 47. Als u drugs gebruikt tijdens het swingen, doet u dit dan samen met uw vaste partner?

- ○ Nooit
- ○ Soms
- ○ Meestal
- ○ Altijd

Swingers, Seksualiteit & Middelengebruik

46. Middelengebruik

* 48. Laat u (of uw partner) de drugs zelf testen voor gebruik?

- Nooit
- Soms
- Meestal
- Altijd

Swingers, Seksualiteit & Middelengebruik

47. Middelengebruik

* 49. Onderstaande stellingen gaan over het testen van drugs.

Wilt u doormiddel van ja of nee aangeven of de stelling voor u van toepassing is?

	Ja	Nee
Ik weet waar ik mijn drugs kan laten testen	○	○
Ik laat elke nieuwe partij drugs zelf testen	○	○
Ik vertrouw erop dat de drugs die ik koop, door de verkoper getest is	○	○
Ik lees altijd de testrapporten van de drugs die ik koop	○	○
Ik vind het belangrijk dat de drugs die ik gebruik, vooraf getest is	○	○

Swingers, Seksualiteit & Middelengebruik

48. Middelengebruik

* 50. Wilt u doormiddel van ja of nee aangeven of de stelling voor u van toepassing is?

	Ja	Nee
Ik heb, voordat ik begon met het gebruiken van drugs, informatie opgezocht over de effecten van drugs	◯	◯
Ik heb mij door swingpartners laten informeren over de effecten van drugs	◯	◯
Ik weet waar ik informatie over de effecten van drugs kan vinden	◯	◯
Informatie over de effecten van drugs is eenvoudig te vinden	◯	◯
Ikzelf of iemand in mijn omgeving is wel eens 'out' gegaan tijdens het swingen	◯	◯
Ik weet hoe ik moet handelen als iemand out gaat of een overdosis heeft gehad	◯	◯

Swingers, Seksualiteit & Middelengebruik

49. Middelengebruik

* 51. Bent u van plan om binnen nu en 12 maanden te stoppen met drugsgebruik tijdens het swingen?

- ◯ Zeker niet
- ◯ Waarschijnlijk niet
- ◯ Misschien
- ◯ Waarschijnlijk wel
- ◯ Zeker wel

Swingers, Seksualiteit & Middelengebruik

50. Middelengebruik

* 52. Wat zijn voor u de belangrijkste redenen om geen drugs te gebruiken?

"Ik gebruik geen drugs, omdat...

	Zeker niet	Waarschijnlijk niet	Misschien	Waarschijnlijk wel	Zeker wel
a ...ik er ziek van kan worden	○	○	○	○	○
b ...de effecten onvoorspelbaar zijn	○	○	○	○	○
c ...ik ‘out’ kan gaan (overdosis)	○	○	○	○	○
d ...ik dan de dag(en) na het swingen vermoeid ben	○	○	○	○	○
e ...drugs verslavend is	○	○	○	○	○
f ...het slecht is voor de gezondheid	○	○	○	○	○
g ...ik er agressief van kan worden	○	○	○	○	○
h ...ik er emotioneel van kan worden	○	○	○	○	○
i ...ik er angstig van kan worden	○	○	○	○	○
j ...ik ervan kan gaan hallucineren	○	○	○	○	○
k ...ik er down/depressief van kan worden (stemmingswisselingen)	○	○	○	○	○
l ...ik dan misschien seksuele handelingen verricht, die ik zonder drugs niet zou verrichten	○	○	○	○	○
m ...ik dan misschien vergeet condooms te gebruiken	○	○	○	○	○
n ...ik slechte ervaringen heb gehad met drugs	○	○	○	○	○
o ...drugs veel geld kost	○	○	○	○	○
p ...mijn partner niet wil dat ik drugs gebruik	○	○	○	○	○
q ...ik geen drugs nodig heb om me prettig te voelen	○	○	○	○	○
r ...ik dan de dag(en) na het swingen minder goed functioneer op het werk	○	○	○	○	○
s ...ik dan de dag(en) na het swingen minder energie heb voor mijn gezin/kinderen	○	○	○	○	○

t ...anders, namelijk:

[ ]

* 53. Welke 3, van bovenstaande effecten, zijn voor u de belangrijkste redenen om geen drugs te gebruiken?

Gebruik hiervoor de letters die voor de effecten staan (bovenstaande vraag)

Swingers, Seksualiteit & Middelengebruik

51. Middelengebruik

* 54. Bent u van plan om binnen nu en 12 maanden drugs te gaan gebruiken tijdens het swingen?

- Zeker niet
- Waarschijnlijk niet
- Misschien
- Waarschijnlijk wel
- Zeker wel

Swingers, Seksualiteit & Middelengebruik

52. Testen op soa

De volgende vragen gaan over het testen op soa

* 55. Hebt u, in de periode dat u swingt, wel eens een geslachtsziekte (soa/HIV) gehad?

- Ja
- Nee

Swingers, Seksualiteit & Middelengebruik

53. Testen op soa

* 56. Hoe vaak heeft u onderstaande geslachtsziekten gehad, in de periode dat u swingt?

	0 keer	1 keer	2 keer	3 keer	4 keer of vaker
Chlamydia	○	○	○	○	○
Genitale wratten	○	○	○	○	○
Genitale Herpes	○	○	○	○	○
Gonorroe	○	○	○	○	○
Hepatitis B	○	○	○	○	○
Syfilis	○	○	○	○	○
HIV	○	○	○	○	○
Schurft	○	○	○	○	○
Schaamluis	○	○	○	○	○
Trichomonas	○	○	○	○	○
Ik weet niet meer welke soa	○	○	○	○	○

Swingers, Seksualiteit & Middelengebruik

54. Testen op soa

* 57. Hebt u in de afgelopen 12 maanden een soa-test gedaan?

○ Ja

○ Nee, maar wel overwogen

○ Nee, ook niet overwogen

Swingers, Seksualiteit & Middelengebruik

55. Testen op soa

* 58. Waar hebt u een soa test gedaan?

- ○ Bij de huisarts
- ○ Bij de GGD
- ○ In het ziekenhuis
- ○ Ik heb een thuistest gedaan
- ○ Anders, namelijk:

[ ]

56. Testen op soa

* 59. Hoe vaak hebt u zich in de afgelopen 12 maanden laten testen?

- ○ 1 keer
- ○ 2 keer
- ○ 3 keer
- ○ Vaker dan 3 keer

Swingers, Seksualiteit & Middelengebruik

57. Testen op soa

* 60. Wat was voor u de belangrijkste reden voor uw meest recente soa-test?

- ○ Ik had onveilige seks en wilde een soa uitsluiten
- ○ Ik doe dit routinematig
- ○ Ik had klachten
- ○ Ik was gewaarschuwd
- ○ Anders, namelijk:

[ ]

Swingers, Seksualiteit & Middelengebruik

58. Testen op soa

* 61. Wilt u bij onderstaande stellingen aangeven of u het eens of oneens bent met de stelling?

	Helemaal oneens	Oneens	Neutraal	Eens	Helemaal eens
Ik vind het belangrijk om mij regelmatig te laten testen op soa	○	○	○	○	○
Ik vind het belangrijk dat mijn swingpartners zich regelmatig laten testen op soa	○	○	○	○	○
Ik vind een soa-test vervelend/ onaangenaam	○	○	○	○	○
Door een soa-test kan ik onveilig vrijen met swingpartners	○	○	○	○	○
Medeswingers vinden dat ik me regelmatig moet laten testen op soa	○	○	○	○	○
Mijn partner vindt dat ik me regelmatig moet laten testen op soa	○	○	○	○	○
De meeste medeswingers laten zich regelmatig testen op soa	○	○	○	○	○
Mijn partner laat zich regelmatig testen op soa	○	○	○	○	○
Het is een plicht je te laten testen op soa	○	○	○	○	○

Swingers, Seksualiteit & Middelengebruik

59. Testen op soa

* 62. Geef aan in hoeverre u het eens bent met onderstaande stelling.

"Ik vind het lastig om me te laten testen omdat...

	Helemaal oneens	Oneens	Neutraal	Eens	Helemaal eens
...ik er tijd voor vrij moet maken	○	○	○	○	○
...ik bang ben voor naalden	○	○	○	○	○
...ik bang ben voor de testuitslag	○	○	○	○	○
...ik bang ben voor de procedure van de soa-test	○	○	○	○	○
...ik er dan voor uit moet komen dat ik swing	○	○	○	○	○
...er kosten aan verbonden zijn	○	○	○	○	○
...ik bekenden kan tegenkomen	○	○	○	○	○
...de openingstijden van testlocaties beperkt zijn	○	○	○	○	○
...mijn vaste partner dit niet mag weten	○	○	○	○	○
...ik vergeet een afspraak te maken voor een soa-test	○	○	○	○	○

Swingers, Seksualiteit & Middelengebruik

60. Testen op soa

* 63. Bent u van plan om binnen nu en 6 maanden een soa-test te doen?

- ○ Zeker niet
- ○ Waarschijnlijk niet
- ○ Misschien
- ○ Waarschijnlijk wel
- ○ Zeker wel

Swingers, Seksualiteit & Middelengebruik

61. Testen op soa

* 64. Bent u van plan zich minimaal 2 keer per jaar te laten testen op soa, zolang u swingt?

- Zeker niet
- Waarschijnlijk niet
- Misschien
- Waarschijnlijk wel
- Zeker wel

Swingers, Seksualiteit & Middelengebruik

62. Testen op soa

* 65. Vul bij de volgende vragen in of deze juist of onjuist zijn.

	Juist	Onjuist
Je merkt dat je een soa hebt doordat je altijd klachten krijgt	◯	◯
Sommige soa gaan vanzelf over	◯	◯
De meeste soa zijn eenvoudig te genezen	◯	◯
Je kunt je gratis laten testen op soa	◯	◯
Je kunt je anoniem laten testen op soa	◯	◯
Testen op soa kan alleen bij de huisarts	◯	◯
Bij een soa-test moet er een wattenstaafje in de urinebuis gebracht worden	◯	◯
Bij een soa-test moet je altijd bloed prikken	◯	◯
Bij een soa-test moet je altijd urine in te leveren	◯	◯

Swingers, Seksualiteit & Middelengebruik

63. Testen op soa

* 66. Wilt u bij onderstaande stellingen aangeven of u het eens of oneens bent met de stelling?

	Helemaal eens	Eens	Neutraal	Oneens	Helemaal oneens
De kans dat ik een soa oploop is erg klein	○	○	○	○	○
Swingers in mijn omgeving hebben weinig geslachtsziekten (soa/HIV)	○	○	○	○	○
Swingers hebben een verhoogd risico op geslachtsziekten (soa/HIV)	○	○	○	○	○
De gevolgen van geslachtsziekten zijn niet ernstig	○	○	○	○	○

Swingers, Seksualiteit & Middelengebruik

64. Testen op soa

* 67. Waar zou u zich het liefste laten testen op soa?

- ○ Bij de GGD
- ○ Bij de huisarts
- ○ In het ziekenhuis
- ○ Thuis (met een thuistest)
- ○ Op plekken waar wordt geswingd (parenclubs, feesten, erotische beurzen)

Swingers, Seksualiteit & Middelengebruik

65. Partnerwaarschuwing

De volgende vragen gaan over partnerwaarschuwing

* 68. Bent u wel eens, in de periode dat u swingt, gewaarschuwd voor een soa?

- ○ Ja, 1 keer
- ○ Ja, 2 keer
- ○ Ja, 3 keer of vaker
- ○ Nee, ik ben nooit gewaarschuwd

Swingers, Seksualiteit & Middelengebruik

66. Partnerwaarschuwing

* 69. Hoe bent u, in de periode dat u swingt, gewaarschuwd voor een soa? Meerdere antwoorden mogelijk.

- [ ] Per telefoon
- [ ] Per sms
- [ ] Per email
- [ ] Face-to-face in een gesprek

Anders, namelijk:

Swingers, Seksualiteit & Middelengebruik

67. Partnerwaarschuwing

* 70. Hebt u zelf wel eens, in de periode dat u swingt, eerdere seksuele partners gewaarschuwd voor een soa?

- ○ Ja, ik heb 1 keer eerdere seksuele partners gewaarschuwd
- ○ Ja, ik heb 2 keer eerdere seksuele partners gewaarschuwd
- ○ Ja, ik heb 3 keer of vaker eerdere seksuele partners gewaarschuwd
- ○ Nee, ik heb nog nooit eerdere seksuele partners gewaarschuwd, mijn vaste partner wel
- ○ Nee, ik heb nog nooit eerdere seksuele partners gewaarschuwd, mijn vaste partner ook niet

Swingers, Seksualiteit & Middelengebruik

68. Partnerwaarschuwing

* 71. Wie hebt u (of uw partner), in de periode dat u swingt, gewaarschuwd voor een soa?

- ○ Alle seksuele contacten
- ○ Bijna alle seksuele contacten
- ○ Een paar seksuele contacten
- ○ Geen enkel seksueel contact

Swingers, Seksualiteit & Middelengebruik

69. Partnerwaarschuwing

* 72. Over welke tijdsperiode hebt u/heeft uw partner eerdere seksuele partners gewaarschuwd?

- ◯ Seksuele contacten van de afgelopen 3 maanden
- ◯ Seksuele contacten van de afgelopen 3 tot 6 maanden
- ◯ Seksuele contacten van de afgelopen 6 tot 12 maanden
- ◯ Seksuele contacten sinds de laatste keer testen

Swingers, Seksualiteit & Middelengebruik

70. Partnerwaarschuwing

* 73. Hoe belangrijk vindt u het om gewaarschuwd te worden voor geslachtsziekten (soa/HIV)?

- ◯ Heel belangrijk
- ◯ Belangrijk
- ◯ Neutraal
- ◯ Onbelangrijk
- ◯ Heel onbelangrijk

Swingers, Seksualiteit & Middelengebruik

71. Partnerwaarschuwing

* 74. Hoe belangrijk vindt u het om zelf recente swingpartners te waarschuwen voor geslachtsziekten (soa/HIV)?

- ◯ Heel belangrijk
- ◯ Belangrijk
- ◯ Neutraal
- ◯ Onbelangrijk
- ◯ Heel onbelangrijk

Swingers, Seksualiteit & Middelengebruik

72. Partnerwaarschuwing

* 75. Wilt u bij onderstaande stellingen aangeven of u het eens of oneens bent met de stelling?

Ik vind het moeilijk om partners te waarschuwen...

	Helemaal oneens	Oneens	Neutraal	Eens	Helemaal eens
...omdat ik bang ben voor de reactie van swingpartners	◯	◯	◯	◯	◯
...omdat ik bang ben buitengesloten te worden	◯	◯	◯	◯	◯
...omdat ik bang ben dat er over mij geroddeld wordt	◯	◯	◯	◯	◯
...omdat ik me zou schamen	◯	◯	◯	◯	◯
...omdat mijn vaste partner niet mag weten dat ik swing	◯	◯	◯	◯	◯
...omdat ik niet altijd contactgegevens van swingpartners heb	◯	◯	◯	◯	◯
...als ik swingpartners lang niet heb gezien	◯	◯	◯	◯	◯
...als swingpartners intieme vrienden zijn	◯	◯	◯	◯	◯

Swingers, Seksualiteit & Middelengebruik

73. Partnerwaarschuwing

* 76. Wilt u bij onderstaande stellingen aangeven in hoeverre deze voor u van toepassing is?

	Zeker niet	Waarschijnlijk niet	Misschien	Waarschijnlijk wel	Zeker wel
Als ik een soa zou hebben, zou ik alle voorgaande partners waarschuwen	○	○	○	○	○
Als ik gewaarschuwd ben voor een soa, zou ik alle voorgaande partners waarschuwen	○	○	○	○	○

Swingers, Seksualiteit & Middelengebruik

74. Demografische gegevens

Tot slot willen u vragen om uw leeftijd, geslacht en hoogst afgeronde opleiding in te vullen.

* 77. Wat is uw geslacht?

○ Man

○ Vrouw

* 78. Wat is uw leeftijd?

[ ]

* 79. Wat is uw hoogst afgeronde opleiding?

○ Basisonderwijs

○ VBO/Mavo

○ Havo/ VWO

○ Middelbaar Beroeps Onderwijs (MBO)

○ Hoger Beroeps Onderwijs (HBO)

○ Wetenschappelijk Onderwijs (WO)

○ Anders, namelijk:

[ ]

80. Hebt u kinderen?

- ◯ Ik heb thuiswonende kinderen
- ◯ Ik heb uitwonende kinderen
- ◯ Ik heb geen kinderen

* 81. Hoe hebt u deze vragenlijst gevonden?

- ◯ Via een GGD website
- ◯ Via een parenclub website
- ◯ Via een swingers website
- ◯ Via een dating website
- ◯ Via een forum
- ◯ Via andere swingers
- ◯ Anders, namelijk:

Swingers, Seksualiteit & Middelengebruik

75. Einde vragenlijst

Geachte deelnemer,

Het online onderzoek naar seksualiteit, middelengebruik en testgedrag bij swingers is afgerond. Daarom is het niet meer mogelijk om de vragenlijst in te vullen.

De resultaten van het onderzoek zullen in december 2011 worden gepubliceerd op de website van de GGD Regio twente.

Swingers, Seksualiteit & Middelengebruik

76. Einde vragenlijst

Dit is het einde van de vragenlijst. Onder de deelnemers worden dinerbonnen t.w.v. € 50,- verloot. Wilt u kans maken op een dinerbon? Vul dan hieronder uw emailadres in. Winnaars krijgen in augustus persoonlijk bericht. Over de uitslag wordt verder niet gecorrespondeerd.
Het emailadres dat u hieronder kunt invullen, wordt alleen gebruikt voor de verloting van de dinerbonnen.

Hartelijk dank voor uw deelname aan het onderzoek!

82. Hebt u nog vragen en/of opmerkingen over dit onderzoek? Dan willen wij u vragen dat hieronder te beschrijven.

Swingers, Seksualiteit & Middelengebruik

77. Einde vragenlijst

Dit is het einde van de vragenlijst
