# Supplementary material for: Sexual behaviour and STI testing among Dutch swingers: A cross-sectional internet based survey performed in 2011 and 2018
Source: PLoS One. 2020 Oct 1;15(10):e0239750. doi: 10.1371/journal.pone.0239750 (PMC7529206; doi:10.1371/journal.pone.0239750)
Supplement: S2 File — (PDF) [file pone.0239750.s002.pdf]

Doet u mee?!

GGD Twente doet onderzoek naar seksualiteit, middelengebruik en testgedrag bij swingers.
Dit doen we om meer inzicht te krijgen in de seksuele gezondheid van mensen die swingen.

Wat bedoelen we met swingen?
Met swingen bedoelen wij dat u, als hetero stel of single, anderen ontmoet voor seksueel contact.

Hoe werkt het?

- **Het invullen van de vragenlijst zal ongeveer een kwartier in beslag nemen.**
- **Het onderzoek is anoniem, dat wil zeggen dat:**

a. u nergens verplicht uw naam hoeft in te vullen;
b. alleen de onderzoekers inzage hebben in de resultaten;
c. de resultaten niet te herleiden zijn naar u.

Wij verzoeken u de vragenlijst zelf in te vullen en niet samen met uw partner.
U kunt slechts één keer deelnemen aan dit onderzoek.

De onderzoeksresultaten zullen rond oktober 2018 gepresenteerd worden op de website van GGD Twente (www.ggdtwente.nl).

Als u de vragenlijst invult, gaan wij er van uit dat u akkoord bent met deelname aan dit onderzoek.

Alvast hartelijk dank!

Swingers, Seksualiteit en Middelengebruik 2018

Swingen

* 1. Bent u ouder dan 18 jaar?

- ◯ Ja
- ◯ Nee

Swingers, Seksualiteit en Middelengebruik 2018

Swingen

* 2. Maakt u deel uit van een man/vrouw stel?

- ◯ Ja
- ◯ Nee

Swingers, Seksualiteit en Middelengebruik 2018

Swingen

* 3. Hebt u, als man/vrouw stel, seksuele contacten met andere stellen en/of singles?

- ◯ Ja
- ◯ Nee

Swingers, Seksualiteit en Middelengebruik 2018

Swingen

* 4. Bent u single?

- ◯ Ja
- ◯ Nee

Swingers, Seksualiteit en Middelengebruik 2018

Swingen

* 5. Hebt u, als single, seksuele contacten met man/vrouw stellen?

- ◯ Ja
- ◯ Nee

Swinggedrag

* 6. Hoeveel keer heeft u in de afgelopen 12 maanden ongeveer geswingd?

- ○ 0 keer per jaar
- ○ 1 keer per jaar
- ○ 2 keer per jaar
- ○ 1 keer per 3 maanden
- ○ 1 keer per maand
- ○ 1 keer per 2 weken
- ○ 1 keer per week
- ○ Vaker dan 1 keer per week

Swingers, Seksualiteit en Middelengebruik 2018

Swinggedrag

* 7. Hoeveel jaren swingt u al?

[ ]

Swingers, Seksualiteit en Middelengebruik 2018

Swinggedrag

* 8. Waar swingt u meestal?

- [ ] Bij mij of een ander thuis
- [ ] In parenclubs
- [ ] In hotels
- [ ] Tijdens (dance/house) feesten
- [ ] Tijdens swingers vakanties
- [ ] Anders, namelijk:

Swingers, Seksualiteit en Middelengebruik 2018

Swinggedrag

* 9. Met wie hebt u tijdens het swingen meestal seksueel contact?

- Meestal met mannen
- Meestal met vrouwen
- Meestal met mannen en vrouwen

Swingers, Seksualiteit en Middelengebruik 2018

Swinggedrag

* 10. Met wie heeft u meestal seks tijdens een swingdate?

(met seks bedoelen we alle vormen van seks: vingeren, aftrekken, pijpen, vaginale seks, anale seks, gebruik van speeltje etc.)

- Alleen met mijn eigen partner
- Met mijn eigen partner én 1 of 2 andere personen
- Met mijn eigen partner én 3 of meer andere personen
- Ik heb geen seks met mijn partner, maar wel met andere personen

Swingers, Seksualiteit en Middelengebruik 2018

Swinggedrag

* 11. Wilt u bij onderstaande stellingen aangeven of deze voor u van toepassing zijn?

	Nooit	Soms	Meestal	Altijd	Niet van toepassing
Ik gebruik een condoom bij vaginale seks met swingpartners	◯	◯	◯	◯	◯
Ik gebruik een condoom bij anale seks met swingpartners	◯	◯	◯	◯	◯
Ik gebruik een condoom/ beflapje bij orale seks met swingpartners	◯	◯	◯	◯	◯
Ik was mijn handen na iedere seksuele handeling	◯	◯	◯	◯	◯
Ik wissel het condoom tussen anaal en vaginaal	◯	◯	◯	◯	◯
Ik wissel het condoom na iedere sekspartner	◯	◯	◯	◯	◯
Ik wissel het condoom bij speeltjes na ieder seksueel contact	◯	◯	◯	◯	◯
Ik reinig speeltjes na ieder seksueel contact	◯	◯	◯	◯	◯

Swingers, Seksualiteit en Middelengebruik 2018

Middelengebruik

De volgende vragen gaan over middelengebruik tijdens het swingen.

* 12. Gebruikt u wel eens potentieverhogende middelen (Kamagra, Viagra, Cialis), tijdens het swingen?

◯ Nooit

◯ Soms

◯ Meestal

◯ Altijd

Swingers, Seksualiteit en Middelengebruik 2018

Middelengebruik

* 13. Gebruikt u wel eens alcohol tijdens het swingen?

- ○ Nooit
- ○ Soms
- ○ Meestal
- ○ Altijd

Swingers, Seksualiteit en Middelengebruik 2018

Middelengebruik

* 14. Hoeveel alcohol drinkt u ongeveer tijdens een swingavond?

- ○ Minder dan 1 glas per avond
- ○ 1 – 2 glazen per avond
- ○ 3 – 4 glazen per avond
- ○ 5 – 6 glazen per avond
- ○ Meer dan 6 glazen per avond

Swingers, Seksualiteit en Middelengebruik 2018

Middelengebruik

15. Gebruikt u wel eens drugs tijdens het swingen?

- ○ Nooit
- ○ Soms
- ○ Meestal
- ○ Altijd

Swingers, Seksualiteit en Middelengebruik 2018

* 16. Welke drugs heeft u ooit gebruikt?

- [ ] Basecoke, crack
- [ ] Cannabis, Hasj, Wiet, Marijuana
- [ ] Crystal Meth, Tina, Ice, Crystal, T, Shabu, Yaba, Shista
- [ ] Cocaïne, Coke, Charlie, Blow, Snow, White
- [ ] GHB, GBL, G, G-tje, Buisje, Tante Gea, Liquid Ecstasy
- [ ] Heroïne, H, Smack, Chiba, Chiva, Bruin
- [ ] Ketamine, K, Special K, Keta, Ket, Vitamin K
- [ ] Lachgas
- [ ] LSD
- [ ] Mephedrone, Meow Meow, 4-MCC, M-Cat, Drone, Miaow, Plant food, MMC-4
- [ ] MDMA, M, Molly
- [ ] MXE Mexxxy, Roflocptr
- [ ] Naphyrone, NRG
- [ ] Paddos, truffels
- [ ] Poppers
- [ ] Ritalin, Concerta, Dexamfetamine (niet als medicatie tegen ADHD)
- [ ] Speeld, Amfetamine, Pep
- [ ] XTC, Ecstasy, Pill, Candy
- [ ] 2-CB
- [ ] 3 MMC
- [ ] 4-FA, 4-FMP, 4 Fluor, 4F, Flava, 4
- [ ] 4-MEC
- [ ] Andere drug(s), namelijk

____

Swingers, Seksualiteit en Middelengebruik 2018

* 17. U heeft aangegeven dat u wel eens drugs heeft gebruikt.
Hoe vaak heeft u in de afgelopen 6 maanden drugs gebruikt voor of tijdens het swingen?

- ◯ Ik heb in de afgelopen 6 maanden helemaal geen drugs gebruikt
- ◯ Ik heb wel drugs gebruikt, maar niet voor of tijdens het swingen
- ◯ Ik heb 1 keer per maand drugs gebruikt
- ◯ Ik heb 2 tot 3 keer per maand drugs gebruikt
- ◯ Ik heb 1 keer per week drugs gebruikt
- ◯ Ik heb meerdere keren per week drugs gebruikt

Swingers, Seksualiteit en Middelengebruik 2018

* 18. Op welke manier heeft u drugs of andere middelen gebruikt in de afgelopen 6 maanden?

- ◯ Slikken (pil, drankje, bommetje (poeder in vloeitje))
- ◯ Laten smelten onder de tong
- ◯ Snuiven
- ◯ Roken
- ◯ Anaal (kont) inbrengen zonder spuit
- ◯ Spuiten in de kont zonder naald (booty bumping)
- ◯ Spuiten met een naald (slammen), zelf of door iemand anders
- ◯ Op een andere manier, namelijk:

Swingers, Seksualiteit en Middelengebruik 2018

* 19. Wat is de reden voor u om drugs te gebruiken tijdens de seks?

U kunt meerdere redenen aanvinken

- [ ] Meer opwinding
- [ ] Minder remming
- [ ] Meer zin in seks
- [ ] Langdurige seks
- [ ] Betere seks
- [ ] Meer seksbeleving
- [ ] Meer zelfvertrouwen
- [ ] Partners lijken aantrekkelijker
- [ ] Meer intimiteit
- [ ] Minder pijn
- [ ] Groepsseks
- [ ] Sociale druk
- [ ] Seksueel experimenteren (FF, BDSM, elektro)
- [ ] Minder zorgen om SOA
- [ ] Iedereen doet het
- [ ] Zucht naar drugs
- [ ] Bij een groep horen
- [ ] Anders, namelijk

Middelengebruik

Ook de volgende vragen gaan over drugsgebruik

* 20. Wilt u aankruisen welke uitspraak voor u het meest van toepassing is?

Het gebruiken van drugs tijdens het swingen vind ik...

	Helemaal oneens	Oneens	Neutraal	Eens	Helemaal eens
Acceptabel	◯	◯	◯	◯	◯
Plezierig	◯	◯	◯	◯	◯
Onverstandig	◯	◯	◯	◯	◯
Gevaarlijk	◯	◯	◯	◯	◯
Spannend	◯	◯	◯	◯	◯
Ongezond	◯	◯	◯	◯	◯

Swingers, Seksualiteit en Middelengebruik 2018

Middelengebruik

* 21. Welke mensen in uw omgeving zouden drugsgebruik goedkeuren/accepteren?

	Zeker niet	Waarschijnlijk niet	Misschien	Waarschijnlijk wel	Zeker wel
Mijn naaste familie	◯	◯	◯	◯	◯
Mijn vrienden	◯	◯	◯	◯	◯
Mijn partner	◯	◯	◯	◯	◯
Mijn swingpartners	◯	◯	◯	◯	◯

Swingers, Seksualiteit en Middelengebruik 2018

Middelengebruik

* 22. Welke mensen in uw omgeving zouden drugsgebruik aanmoedigen/stimuleren?

	Zeker niet	Waarschijnlijk niet	Misschien	Waarschijnlijk wel	Zeker wel
Mijn naaste familie	○	○	○	○	○
Mijn vrienden	○	○	○	○	○
Mijn partner	○	○	○	○	○
Mijn swingpartners	○	○	○	○	○

Middelengebruik

* 23. Wat was voor u de belangrijkste reden om te starten met drugsgebruik?

- ○ Ik werd nieuwsgierig omdat ik mensen in mijn omgeving zag gebruiken of hierover hoorde
- ○ Ik kreeg drugs aangeboden
- ○ Ik werd overgehaald om drugs te gebruiken
- ○ Anders, namelijk:

Swingers, Seksualiteit en Middelengebruik 2018

Middelengebruik

* 24. Als u drugs gebruikt tijdens het swingen, welke POSITIEVE effecten ervaart u dan?

U kunt meerdere effecten aanvinken

“Als ik drugs gebruik, dan...

- [ ] ...heb ik meer energie en kan ik langer doorgaan
- [ ] ...ben ik meer alert
- [ ] ...kan ik hardere seksuele handeling verrichten en/of laten verrichten
- [ ] ...ervaar ik een liefdevol/intiem gevoel
- [ ] ...ervaar ik een rustig / ontspannen gevoel
- [ ] ...ervaar ik een positief / gelukkig gevoel
- [ ] ...vergeet ik mijn problemen
- [ ] ...ben ik meer seksueel opgewonden
- [ ] ...ervaar ik minder remmingen / voel ik mij vrijer
- [ ] ...kan ik meer genieten omdat het mijn orgasme uitstelt
- [ ] ...kan ik meer genieten omdat het orgasme intenser wordt
- [ ] ...beleef ik licht en geluid intenser
- [ ] ...ben ik mij minder bewust van tijd en plaats
- [ ] ...leg ik makkelijker contacten
- [ ] ...raak ik makkelijker andere mensen aan en/of laat ik mij makkelijker aanraken
- [ ] ...voel ik mij, de dag(en) na het swingen prettiger
- [ ] ...anders, namelijk:

Middelengebruik

* 25. Als u drugs gebruikt tijdens het swingen, welke NEGATIEVE effecten ervaart u dan?

U kunt meerdere effecten aanvinken.

“Als ik drugs gebruik, dan...

- [ ] ...voel ik mij misselijk
- [ ] ...krijg ik hoofdpijn
- [ ] ...ben ik, de dagen na het swingen, vermoeid
- [ ] ...heb ik steeds meer drugs nodig voor hetzelfde effect
- [ ] ...heb ik, de dagen na het swingen, last van slapeloosheid
- [ ] ...kan ik verslaafd raken
- [ ] ...schaadt dit mijn gezondheid
- [ ] ...voel ik mij, de dagen na het swingen, down/depressief
- [ ] ...vind ik het niet prettig meer om seks te hebben zonder drugs
- [ ] ...functioneer ik, de dagen na het swingen, minder goed op mijn werk
- [ ] ...heb ik, de dagen na het swingen, minder energie voor mijn gezin/kinderen
- [ ] ...anders, namelijk:

Middelengebruik

* 26. Als u drugs gebruikt tijdens het swingen, doet u dit dan samen met uw vaste partner?

- ○ Nooit
- ○ Soms
- ○ Meestal
- ○ Altijd

Swingers, Seksualiteit en Middelengebruik 2018

Middelengebruik

* 27. Laat u (of uw partner) de drugs zelf testen voor gebruik?

- ◯ Nooit
- ◯ Soms
- ◯ Meestal
- ◯ Altijd

Swingers, Seksualiteit en Middelengebruik 2018

Middelengebruik

* 28. Onderstaande stellingen gaan over het testen van drugs.

Wilt u doormiddel van ja of nee aangeven of de stelling voor u van toepassing is?

	Ja	Nee
Ik weet waar ik mijn drugs kan laten testen	◯	◯
Ik laat elke nieuwe partij drugs zelf testen	◯	◯
Ik vertrouw erop dat de drugs die ik koop, door de verkoper getest is	◯	◯
Ik lees altijd de testrapporten van de drugs die ik koop	◯	◯
Ik vind het belangrijk dat de drugs die ik gebruik, vooraf getest is	◯	◯

Swingers, Seksualiteit en Middelengebruik 2018

Middelengebruik

* 29. Wilt u doormiddel van ja of nee aangeven of de stelling voor u van toepassing is?

	Ja	Nee
Ik heb, voordat ik begon met het gebruiken van drugs, informatie opgezocht over de effecten van drugs	○	○
Ik heb mij door swingpartners laten informeren over de effecten van drugs	○	○
Ik weet waar ik informatie over de effecten van drugs kan vinden	○	○
Informatie over de effecten van drugs is eenvoudig te vinden	○	○
Ikzelf of iemand in mijn omgeving is wel eens 'out' gegaan tijdens het swingen	○	○
Ik weet hoe ik moet handelen als iemand out gaat of een overdosis heeft gehad	○	○

Swingers, Seksualiteit en Middelengebruik 2018

Middelengebruik

* 30. Bent u van plan om binnen nu en 12 maanden te stoppen met drugsgebruik tijdens het swingen?

- ○ Zeker niet
- ○ Waarschijnlijk niet
- ○ Misschien
- ○ Waarschijnlijk wel
- ○ Zeker wel

Swingers, Seksualiteit en Middelengebruik 2018

Middelengebruik

31. Wat zijn voor u de belangrijkste redenen om geen drugs te gebruiken?

U kunt meerdere redenen aanvinken.

"Ik gebruik geen drugs, omdat...

- ☐ ...ik er ziek van kan worden
- ☐ ...de effecten onvoorspelbaar zijn
- ☐ ...ik 'out' kan gaan (overdosis)
- ☐ ...ik dan de dag(en) na het swingen vermoeid ben
- ☐ ...drugs verslavend is
- ☐ ...het slecht is voor de gezondheid
- ☐ ...ik er agressief van kan worden
- ☐ ...ik er emotioneel van kan worden
- ☐ ...ik er angstig van kan worden
- ☐ ...ik ervan kan gaan hallucineren
- ☐ ...ik er down/depressief van kan worden (stemmingswisselingen)
- ☐ ...ik dan misschien seksuele handelingen verricht, die ik zonder drugs niet zou verrichten
- ☐ ...ik dan misschien vergeet condooms te gebruiken
- ☐ ...ik slechte ervaringen heb gehad met drugs
- ☐ ...drugs veel geld kost
- ☐ ...mijn partner niet wil dat ik drugs gebruik
- ☐ ...ik geen drugs nodig heb om me prettig te voelen
- ☐ ...ik dan de dag(en) na het swingen minder goed functioneer op het werk
- ☐ ...ik dan de dag(en) na het swingen minder energie heb voor mijn gezin/kinderen
- ☐ ...anders, namelijk:

Swingers, Seksualiteit en Middelengebruik 2018

Middelengebruik

* 32. Bent u van plan om binnen nu en 12 maanden drugs te gaan gebruiken tijdens het swingen?

- Zeker niet
- Waarschijnlijk niet
- Misschien
- Waarschijnlijk wel
- Zeker wel

Swingers, Seksualiteit en Middelengebruik 2018

Middelengebruik

* 33. Swingt u met mensen die drugs gebruiken?

- Nooit
- Soms
- Meestal
- Altijd

Swingers, Seksualiteit en Middelengebruik 2018

34. Hoe vaak heeft u een beloning gehad voor seks in de afgelopen 6 maanden? Denk aan geld of goederen.

- Nooit
- Eenmaal
- Meerdere malen

Swingers, Seksualiteit en Middelengebruik 2018

* 35. Wat heeft u gekregen als beloning voor seks?

- Geld
- Spullen
- Drugs
- Iets anders, namelijk

Testen op soa's

De volgende vragen gaan over het testen op soa's

* 36. Heeft u, in de periode dat u swingt, wel eens een seksueel overdraagbare aandoening gehad (soa)?

Bijvoorbeeld chlamydia, gonorroe, syfilis, hepatitis B, herpes, genitale wratjes, schurft, schaamluis, trichomonas of HIV?

- Ja
- Nee

Swingers, Seksualiteit en Middelengebruik 2018

Testen op soa

37. Welke van de onderstaande soa's heeft u gehad?

Het gaat om de soa's die u heeft opgelopen tijdens het swingen.

- ☐ Chlamydia
- ☐ Genitale wratten
- ☐ Genitale Herpes
- ☐ Gonorroe
- ☐ Hepatitis B
- ☐ Syfilis
- ☐ HIV
- ☐ Schurft
- ☐ Schaamluis
- ☐ Trichomonas
- ☐ Ik weet niet meer welke soa

Testen op soa

* 38. Heeft u in de afgelopen 12 maanden een soa-test gedaan?

- ◯ Ja
- ◯ Nee, maar wel overwogen
- ◯ Nee, ook niet overwogen

Swingers, Seksualiteit en Middelengebruik 2018

Testen op soa

* 39. Waar heeft u een soa test gedaan?

- ◯ Bij de huisarts
- ◯ Bij de GGD
- ◯ In het ziekenhuis
- ◯ Ik heb een thuistest gedaan
- ◯ Anders, namelijk:

Swingers, Seksualiteit en Middelengebruik 2018

Testen op soa

* 40. Hoe vaak heeft u zich in de afgelopen 12 maanden laten testen?

- ◯ 1 keer
- ◯ 2 keer
- ◯ 3 keer
- ◯ Vaker dan 3 keer

Swingers, Seksualiteit en Middelengebruik 2018

Testen op soa

* 41. Wat was voor u de belangrijkste reden voor uw meest recente soa-test?

- ◯ Ik had onveilige seks en wilde een soa uitsluiten
- ◯ Ik doe dit routinematig
- ◯ Ik had klachten
- ◯ Ik was gewaarschuwd
- ◯ Anders, namelijk:

Swingers, Seksualiteit en Middelengebruik 2018

Testen op soa

* 42. Wilt u bij onderstaande stellingen aangeven of u het eens of oneens bent met de stelling?

	Helemaal oneens	Oneens	Neutraal	Eens	Helemaal eens
Ik vind het belangrijk om mij regelmatig te laten testen op soa	○	○	○	○	○
Ik vind het belangrijk dat mijn swingpartners zich regelmatig laten testen op soa	○	○	○	○	○
Ik vind een soa-test vervelend/ onaangenaam	○	○	○	○	○
Door een soa-test kan ik onveilig vrijen met swingpartners	○	○	○	○	○
Medeswingers vinden dat ik me regelmatig moet laten testen op soa	○	○	○	○	○
Mijn partner vindt dat ik me regelmatig moet laten testen op soa	○	○	○	○	○
De meeste medeswingers laten zich regelmatig testen op soa	○	○	○	○	○
Mijn partner laat zich regelmatig testen op soa	○	○	○	○	○
Het is een plicht je te laten testen op soa	○	○	○	○	○

Testen op soa

* 43. Geef aan in hoeverre u het eens bent met onderstaande stelling.

"Ik vind het lastig om me te laten testen omdat...

	Helemaal oneens	Oneens	Neutraal	Eens	Helemaal eens
...ik er tijd voor vrij moet maken	○	○	○	○	○
...ik bang ben voor naalden	○	○	○	○	○
...ik bang ben voor de testuitslag	○	○	○	○	○
...ik bang ben voor de procedure van de soa-test	○	○	○	○	○
...ik er dan voor uit moet komen dat ik swing	○	○	○	○	○
...er kosten aan verbonden zijn	○	○	○	○	○
...ik bekenden kan tegenkomen	○	○	○	○	○
...de openingstijden van testlocaties beperkt zijn	○	○	○	○	○
...mijn vaste partner dit niet mag weten	○	○	○	○	○
...ik vergeet een afspraak te maken voor een soa-test	○	○	○	○	○

Swingers, Seksualiteit en Middelengebruik 2018

Testen op soa

* 44. Bent u van plan om binnen nu en 6 maanden een soa-test te doen?

- ○ Zeker niet
- ○ Waarschijnlijk niet
- ○ Misschien
- ○ Waarschijnlijk wel
- ○ Zeker wel

Swingers, Seksualiteit en Middelengebruik 2018

Testen op soa

* 45. Vul bij de volgende vragen in of deze juist of onjuist zijn.

	Juist	Onjuist
Je merkt dat je een soa hebt doordat je altijd klachten krijgt	◯	◯
Sommige soa gaan vanzelf over	◯	◯
De meeste soa zijn eenvoudig te genezen	◯	◯
Je kunt je gratis laten testen op soa	◯	◯
Je kunt je anoniem laten testen op soa	◯	◯
Testen op soa kan alleen bij de huisarts	◯	◯
Bij een soa-test moet er een wattenstaafje in de urinebuis gebracht worden	◯	◯
Bij een soa-test moet je altijd bloed prikken	◯	◯
Bij een soa-test moet je altijd urine in te leveren	◯	◯

Testen op soa

* 46. Wilt u bij onderstaande stellingen aangeven of u het eens of oneens bent met de stelling?

	Helemaal eens	Eens	Neutraal	Oneens	Helemaal oneens
De kans dat ik een soa oploop is erg klein	◯	◯	◯	◯	◯
Swingers in mijn omgeving hebben weinig geslachtsziekten (soa/HIV)	◯	◯	◯	◯	◯
Swingers hebben een verhoogd risico op geslachtsziekten (soa/HIV)	◯	◯	◯	◯	◯
De gevolgen van geslachtsziekten zijn niet ernstig	◯	◯	◯	◯	◯

Swingers, Seksualiteit en Middelengebruik 2018

Testen op soa

* 47. Waar zou u zich het liefste laten testen op soa?

- Bij de GGD
- Bij de huisarts
- In het ziekenhuis
- Thuis (met een thuistest)
- Op plekken waar wordt geswingd (parenclubs, feesten, erotische beurzen)

Partnerwaarschuwing

De volgende vragen gaan over partnerwaarschuwing

* 48. Bent u wel eens, in de periode dat u swingt, gewaarschuwd voor een soa?

- Nee, nog nooit
- Ja, 1 keer
- Ja, 2 keer
- Ja, 3 keer of vaker

Swingers, Seksualiteit en Middelengebruik 2018

Partnerwaarschuwing

* 49. Hoe bent u, in de periode dat u swingt, gewaarschuwd voor een soa? Meerdere antwoorden mogelijk.

- Per telefoon: app/sms of telefoongesprek
- Per email
- Face-to-face in een gesprek

Anders, namelijk:

Swingers, Seksualiteit en Middelengebruik 2018

Partnerwaarschuwing

* 50. Heeft u zelf wel eens sekspartners gewaarschuwd voor een soa?

- ◯ Ja, 1 keer
- ◯ Ja, 2 keer
- ◯ Ja, 3 keer of vaker
- ◯ Nee, ik heb nog nooit sekspartners gewaarschuwd, mijn vaste partner wel
- ◯ Nee, ik heb nog nooit sekspartners gewaarschuwd, mijn vaste partner ook niet

Partnerwaarschuwing

* 51. U heeft wel eens en swingpartner gewaarschuwd voor een soa. Wie heeft u precies gewaarschuwd?

- ◯ Alle seksuele contacten
- ◯ Bijna alle seksuele contacten
- ◯ Een paar seksuele contacten
- ◯ Geen enkel seksueel contact

Swingers, Seksualiteit en Middelengebruik 2018

Partnerwaarschuwing

* 52. U heeft wel eens en swingpartner gewaarschuwd voor een soa.

Kunt u aangeven welke stelling voor u van toepassing is?

- ◯ Ik heb mijn sekscontacten van de afgelopen 3 maand gewaarschuwd
- ◯ Ik heb mijn sekscontacten van de afgelopen 6 maanden gewaarschuwd
- ◯ Ik heb mijn sekscontacten van de afgelopen 12 maanden gewaarschuwd
- ◯ Ik heb alle mensen gewaarschuwd waar ik sinds de laatste soa-test seks mee heb gehad.

Swingers, Seksualiteit en Middelengebruik 2018

Partnerwaarschuwing

* 53. Hoe belangrijk vindt u het om gewaarschuwd te worden voor soa's?

- Heel belangrijk
- Belangrijk
- Neutraal
- Onbelangrijk
- Heel onbelangrijk

Partnerwaarschuwing

* 54. Stel u heeft tijdens het swingen een soa opgelopen. Hoe belangrijk vindt u het dan om swingpartners te waarschuwen?

- Heel belangrijk
- Belangrijk
- Neutraal
- Onbelangrijk
- Heel onbelangrijk

Partnerwaarschuwing

* 55. Wilt u bij onderstaande stellingen aangeven of u het eens of oneens bent met de stelling?

Ik vind het moeilijk om partners te waarschuwen...

	Helemaal oneens	Oneens	Neutraal	Eens	Helemaal eens
...omdat ik bang ben voor de reactie van swingpartners	○	○	○	○	○
...omdat ik bang ben buitengesloten te worden	○	○	○	○	○
...omdat ik bang ben dat er over mij geroddeld wordt	○	○	○	○	○
...omdat ik me zou schamen	○	○	○	○	○
...omdat mijn vaste partner niet mag weten dat ik swing	○	○	○	○	○
...omdat ik niet altijd contactgegevens van swingpartners heb	○	○	○	○	○
...als ik swingpartners lang niet heb gezien	○	○	○	○	○
...als swingpartners intieme vrienden zijn	○	○	○	○	○

Partnerwaarschuwing

* 56. Wilt u bij onderstaande stellingen aangeven in hoeverre deze voor u van toepassing is?

	Zeker niet	Waarschijnlijk niet	Misschien	Waarschijnlijk wel	Zeker wel
Als ik een soa zou hebben, zou ik alle voorgaande partners waarschuwen	○	○	○	○	○
Als ik gewaarschuwd ben voor een soa, zou ik alle voorgaande partners waarschuwen	○	○	○	○	○

Demografische gegevens

Tot slot willen u vragen om uw leeftijd, geslacht en hoogst afgeronde opleiding in te vullen.

* 57. Wat is uw geslacht?

○ Man

○ Vrouw

* 58. Wat is uw leeftijd?

* 59. Wat is uw hoogst afgeronde opleiding?

- Basisonderwijs
- VBO/Mavo
- Havo/ VWO
- Middelbaar Beroeps Onderwijs (MBO)
- Hoger Beroeps Onderwijs (HBO)
- Wetenschappelijk Onderwijs (WO)
- Anders, namelijk:

60. Heeft u kinderen?

- Ik heb thuiswonende kinderen
- Ik heb uitwonende kinderen
- Ik heb geen kinderen

* 61. Hoe heeft u deze vragenlijst gevonden?

- Via een GGD website
- Via een parenclub website
- Via een swingers website
- Via een dating website
- Via een forum
- Via andere swingers
- Anders, namelijk:

Swingers, Seksualiteit en Middelengebruik 2018

Einde vragenlijst

Alleen personen van 18 jaar en ouder kunnen deelnemen aan dit onderzoek. Daarom wordt deze vragenlijst nu afgesloten.

Swingers, Seksualiteit en Middelengebruik 2018

Einde vragenlijst

Alleen stellen die seks hebben met andere stellen en/of singles vallen binnen de criteria van dit onderzoek. Daarom wordt de vragenlijst nu afgesloten.

Swingers, Seksualiteit en Middelengebruik 2018

Alleen singles die seks hebben met andere stellen vallen binnen de criteria van dit onderzoek. Daarom wordt de vragenlijst nu afgesloten.

Swingers, Seksualiteit en Middelengebruik 2018

Alleen als u het afgelopen jaar tenminste 1 keer heeft geswingd valt u binnen de criteria van dit onderzoek. Daarom wordt de vragenlijst nu afgesloten.

Swingers, Seksualiteit en Middelengebruik 2018

Einde vragenlijst

Dit is het einde van de vragenlijst! Heel hartelijk dank voor uw deelname aan het onderzoek!

62. Heeft u nog vragen en/of opmerkingen over dit onderzoek? Dan willen wij u vragen dat hieronder te beschrijven.
Verwacht u van ons een antwoord? Vul dan uw e-mailadres of telefoonnummer in.

Swingers, Seksualiteit en Middelengebruik 2018

Kans maken op een dinerbon!

Onder de deelnemers verloten wij vijf dinerbonnen t.w.v. € 50,-.
Wilt u kans maken op een dinerbon? klik dan HIER

Swingers, Seksualiteit en Middelengebruik 2018

Einde vragenlijst

De vragenlijst wordt nu afgesloten.
